# The Screw Tightens

**Published:** 1896-09

**Authors:** 


THE SCREW TIGHTENS.—Can we stand one more turn? The pressure brought to bear upon the naughty, incorrigible editor of the "Red Back" who has the temerity, shall we say? or the audacity, or, the what shall we call it?—the courage to oppose the proposed degradation of medicine by the alliance with homeopaths — is tremendous. We are ridiculed, reviled, scoffed at, called Don Quixote—and that too by a regular Sancho Panza, besought, pleaded with, bullied, threatened, all because we will not fall in line with the proposition. In the Medical Windmill,—beg pardon—*Texas Medical News* of August, in sympathy and accord with the tenets and advocacy of that publication, is a lettter from Dr. E. Mussina, a respectable homeopath of Austin, who chimes "me too" to McLaughlin's pleas;—a regular "how we apples do swim" kind of a letter, very eager for a leveling of all distinctions (leveling on the part of medicine; elevating to an unmerited dignity the several pathies.) We will have to give in, I suppose, or give over our obduracy now that we are attacked in this way. Can we stand the pressure? In a letter to the *News*—under the caption "Misapprehension will Make Enemies where Reasoning might Make Friends," this disciple of Hahnemann says—amongst a lot of other things—not worth mentioning:

"Ye doctors! ye doctors! What be the matter with you? How can the layity [*sic*] respect the true dignity of our profession [our profession is good—and in light of the proposed bill—

appropriate] if we insist upon having adverse interests and conceptions of the common wellfare? We have just read in the July number of the TEXAS MEDICAL JOURNAL that the Texas State Medical Association has passed a bill draft to be recommended to the next legislature for a general law regulating the practice of medicine. The editor of the 'Red Back' will not allow himself to become modernized."

That's it, is it? To become modernized, the writer means that we should *homologate*,—merge medicine and homeopathy, and be hail fellows with every pathy—for the common good! Well, we will be content to remain old fashioned for a while longer—if to be not modern means to stand out for the honor and dignity of the profession of medicine.

The *Texas Medical News* ought to be happy now that it has secured this endorsement by a representative homo, who spells "laity" "layity." Humph!

